# Sloshing Measurements inside a Liquid Hydrogen Tank with External-Heating-Type MgB_2_ Level Sensors during Marine Transportation by the Training Ship Fukae-Maru

**DOI:** 10.3390/s18113694

**Published:** 2018-10-30

**Authors:** Kazuma Maekawa, Minoru Takeda, Yuuki Miyake, Hiroaki Kumakura

**Affiliations:** 1 Graduate School of Maritime Sciences, Kobe University, 5-1-1 Fukaeminami-machi, Higashinada-ku, Kobe 658-0022, Japan; takeda@maritime.kobe-u.ac.jp (M.T.); 170w529w@stu.kobe-u.ac.jp (Y.M.); 2 National Institute for Materials Science, 1-2-1 Sengen, Tsukuba 305-0047, Japan; KUMAKURA.Hiroaki@nims.go.jp

**Keywords:** MgB_2_ liquid hydrogen level sensor, sloshing, marine transportation

## Abstract

Recently, a project was initiated in Japan to transport a large amount of liquid hydrogen (LH_2_) from Australia to Japan by sea. It is important to understand the sloshing and boil-off that are likely to occur inside an LH_2_ tank during marine transportation by ship, but such characteristics are yet to be experimentally clarified. To do so, we combined the liquid level detected by five 500 mm long external-heating-type magnesium diboride (MgB_2_) level sensors with synchronous measurements of temperature, pressure, ship motion, and acceleration during a zigzag maneuver. During this zigzag maneuver, the pressure of gaseous hydrogen (GH_2_) in the small LH_2_ tank increased to roughly 0.67 MPaG/h, and the temperature of the GH_2_ in the small LH_2_ tank increased at the position of gaseous hydrogen at roughly 1.0 K/min when the maximum rolling angle was 5°; the average rolling and liquid-oscillation periods were 114 and 118 s, respectively, as detected by the MgB_2_ level sensors, which therefore detected a long-period LH_2_ wave due to the ship’s motion.

## 1. Introduction

In 2015, the 21st session of the Conference of the Parties (COP21) to the United Nations Framework Convention on Climate Change was convened in Paris, France. A goal was agreed on for the parties to the Paris Agreement to reduce their greenhouse gas emissions in the post-2020 period. Against this background, hydrogen has been attracting immense attention as a medium for solving global environmental problems and avoiding a future energy crisis, because only water is produced when hydrogen chemically reacts with oxygen. Moreover, hydrogen is highly attractive as a secondary energy medium for renewable energies such as solar and wind power.

In Japan, a country with insufficient energy resources, it is very important to establish where and how to produce hydrogen if hydrogen energy is to become widespread. Currently, hydrogen gas is produced using brown coal from Australia. This hydrogen gas is then liquefied on site because liquid hydrogen (LH_2_: 20 K) is roughly 800 times denser than gaseous hydrogen (GH_2_: 300 K, 0.1 MPa). Thus, from now until 2020, large quantities of LH_2_ will be transported by sea from Australia to Kobe, Japan by a cargo ship equipped with two 1250 m^3^ LH_2_ tanks [1].

The characteristics of LH_2_ sloshing and boil-off that occur during marine transportation are yet to be experimentally clarified, despite a similar phenomenon occurring inside tanks of liquefied natural gas (LNG, 112 K). The boil-off is generated by natural heat input in the stationary state, but boil-off by sloshing inside the LH_2_ tank is added during marine transportation. Sloshing LH_2_ does not damage the tank because the storage weight is much smaller than that of LNG. However, the amount of LH_2_ evaporation is 10 times higher than that of LNG because the amount of boil-off gas (BOG) increases as heat is exchanged with the inner wall of the LH_2_ tank when sloshing occurs inside it [1]. To reduce the amount of BOG, it is important to study the sloshing characteristics of an LH_2_ tank.

The external-heating-type superconducting magnesium diboride (MgB_2_) level sensor for LH_2_ is highly linear and has high resolution, good reproducibility, and good static and dynamic level-detecting characteristics [2,3,4,5,6,7,8,9,10,11]. Moreover, its response time to variations of LH_2_ level is around 0.1 s, and the difference between the level optically read and that detected by MgB_2_ level sensors is around 5 mm at a heater input of 9 W [12,13]. Thus, multiple MgB_2_ level sensors have been installed inside an LH_2_ tank to simultaneously measure the liquid level, and these sensors are expected to clarify the sloshing inside an LH_2_ tank.

The purpose of the present study was to use five 500 mm long external-heating-type MgB_2_ level sensors to understand the sloshing that occurred inside the LH_2_ optical cryostat (small LH_2_ tank) during marine transportation by the training ship Fukae-maru. Previous papers have outlined the first experiments conducted on LH_2_ transport by the Fukae-maru and have presented qualitative results on the rates of temperature and pressure increase inside its LH_2_ tank, but with no measurements of liquid level [14]. In the present paper, we discuss (i) the relationship between the liquid-surface oscillation detected by five MgB_2_ level sensors and the rates of temperature and pressure increase inside the small LH_2_ tank; (ii) the relationship between the rolling and pitching angles and the acceleration of the ship; and (iii) the relationship between the liquid-surface oscillation detected by the five MgB_2_ level sensors and the rolling and pitching periods of the ship during a zigzag maneuver.

## 2. External-Heating-Type MgB_2_ Level Sensor

**Figure 1:**
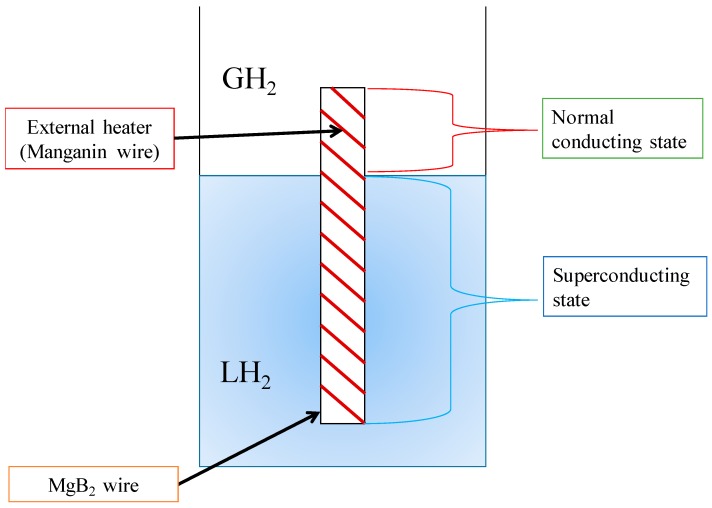
Principle of the external-heating-type MgB_2_ level sensor.

Figure 1 shows the measurement principle of the external-heating-type MgB_2_ level sensor. A superconducting level sensor utilizes differing electrical resistance *R* between the liquid phase (*R* = 0) and the vapor phase (*R* > 0), meaning that the liquid level can be obtained by measuring the overall electrical resistance of the MgB_2_ level sensor. However, when exposed to the vapor phase, the sensor is cooled by the evaporated gas, and part of it becomes superconducting. Thus, an external heater is wound in a spiral around the MgB_2_ wire. The MgB_2_ wire used in this experiment was 0.32 mm in diameter and reinforced by a CuNi (7:3) sheath. It was fabricated in situ using the powder-in-tube method with a heat treatment of 1 h at 873.15 K. To reduce critical temperature *T*_c_ of the MgB_2_ wire, 10% SiC was added as an impurity to the MgB_2_ core; as a result, *T*_c_ was around 32 K. A polyester-coated Manganin wire of 0.2 mm diameter was wound in a spiral around the MgB_2_ wire with a pitch of 2 mm for use as an external heater. For this experiment, we manufactured five 500 mm long MgB_2_ level sensors (denoted as A1, A2, B1, B2, and C), which were cut from three 1.7 m long MgB_2_ wires (wires A, B, and C). After that, we examined their static and dynamic level-detecting characteristics. The results showed that performance variation among the MgB_2_ sensors caused by individual differences in the wire rods was minimal, and that these MgB_2_ level sensors have good reproducibility [10,11,12,13].

## 3. Apparatus and Method

**Figure 2:**
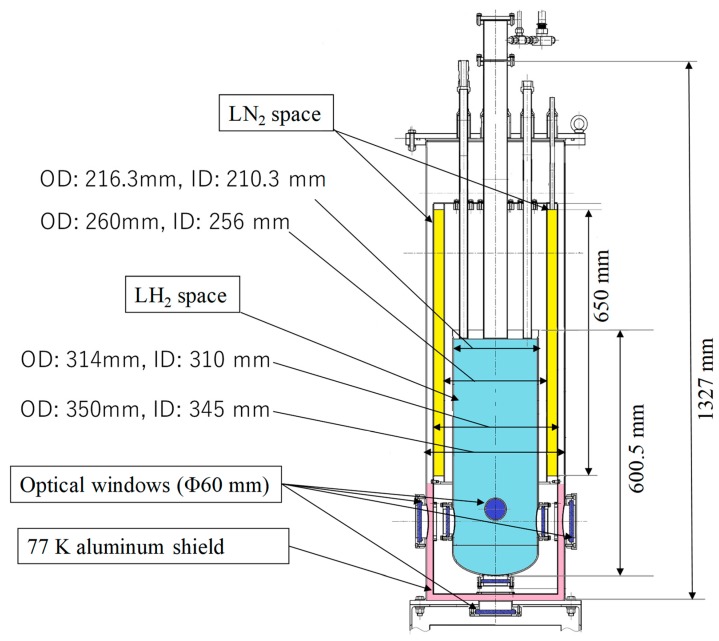
Schematic diagram of the experimental apparatus.

**Figure 3:**
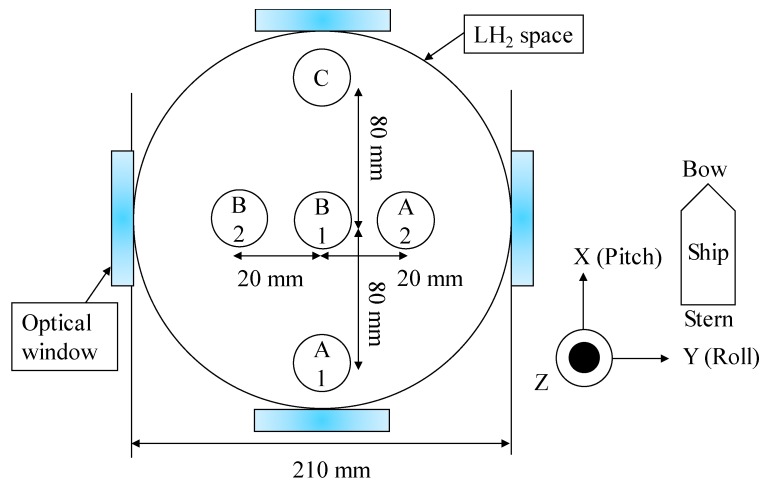
Layout of the five MgB_2_ level sensors with a diameter of 0.32 mm.

Figure 2 shows a schematic diagram of the LH_2_ optical cryostat (small LH_2_ tank), which comprises a vacuum jacket, an LH_2_ space (20 L), a liquid nitrogen (LN_2_) space (15 L), a 77 K aluminum shield, and five optical windows. The optical cryostat is 1327 mm tall, and the optical windows are made of Pyrex glass and have an effective diameter of 60 mm. The natural heat-input power to the LH_2_ space was found experimentally to be roughly 0.72 W, and that to the LN_2_ space was roughly 5.8 W when all optical windows were covered with aluminum caps. Figure 3 shows the layout of the five MgB_2_ level sensors. The bow direction of the ship was equal to the pitch direction, as shown in Figure 3. Sensors B2 and A2 were positioned to detect the liquid-surface oscillation in the roll direction, A1 and C were positioned to detect the liquid-surface oscillation in the pitch direction, and B1 was positioned to detect the liquid-surface oscillation at the center of the small LH_2_ tank. In addition, all five sensors were fixed with a stainless steel plate and the glass fiber-reinforced plastics (GFRP) rod.

**Figure 4:**
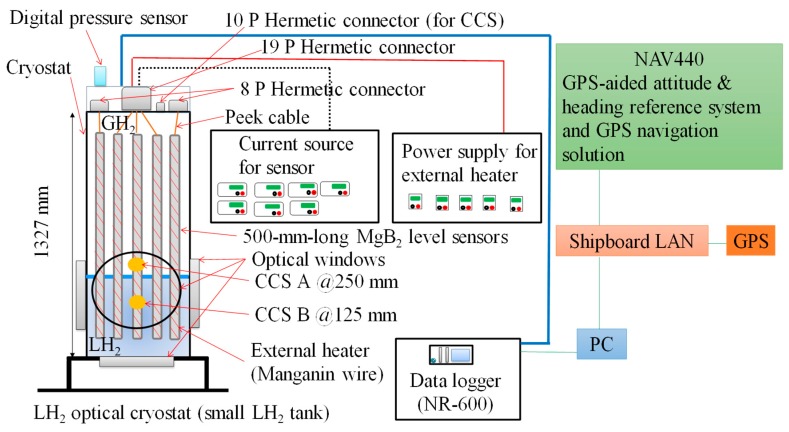
Measurement system.

Figure 4 shows the measurement system. It consists of an optical cryostat (a small LH_2_ tank), five MgB_2_ level sensors, two carbon ceramic temperature sensors (CCSs), a digital pressure sensor, current sources for the level sensors and CCSs, a power supply for the external heaters of the level sensors, a data logger (NR-600; Keyence, Osaka, Japan), a GPS-aided microelectromechanical inertial system (NAV440; Memsic, Andover, MA, USA), and a PC. The A and B CCSs were attached to the level-sensor support at distances of 250 and 125 mm, respectively, from the bottom of level Sensor B1. Data regarding liquid level, temperature, and pressure inside the cryostat were obtained by the data logger, and data regarding ship motion (in the form of accelerations in the X, Y, and Z directions) were obtained by the inertial system through a shipboard local area network. All the data were collected synchronously by the PC in conjunction with the GPS clock.

**Figure 5:**
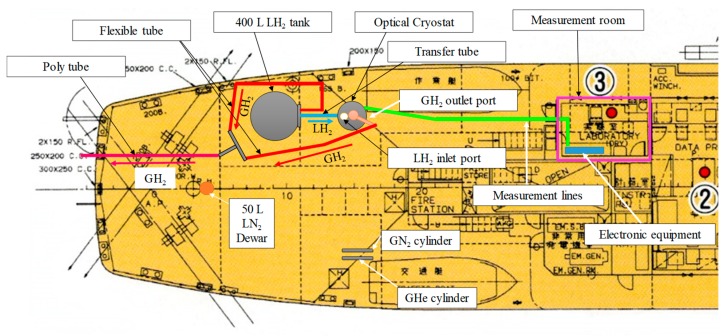
Setup of the experimental apparatus.

**Figure 6:**
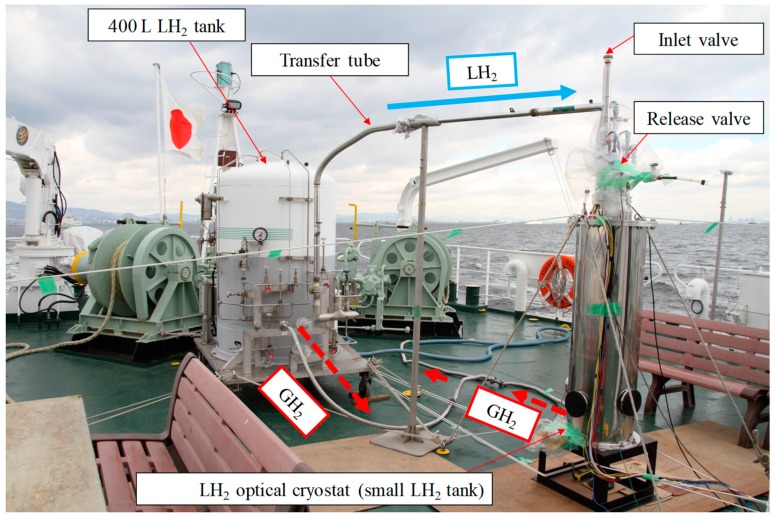
Photograph of the experimental setup on the afterdeck.

Figure 5 shows the setup of the experimental apparatus. Figure 6 shows a photograph of the experimental setup on the afterdeck of the training ship Fukae-maru, which is 50 m long and has a gross weight of 449 tons. All electronic equipment was placed in the onboard measurement room to protect it from possible explosion. In addition, all optical windows were covered with aluminum caps during the marine-transportation test.

**Figure 7:**
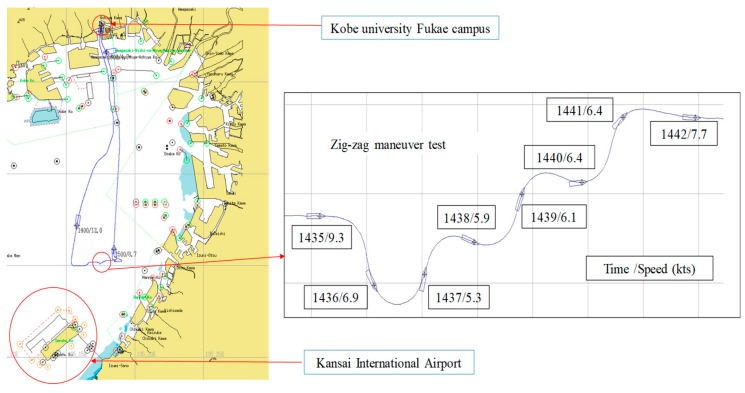
Track chart of the Fukae-maru inside Osaka Bay.

Table 1 provides a time chart of the experimental processes conducted in Osaka Bay on February 2, 2017, and Figure 7 shows the corresponding track chart of the Fukae-maru. In Table 1, test numbers ①–⑥ refer to the experimental processes: ①–③ denote rapid depressurizations of the small LH_2_ tank with a release valve, ④ denotes drifting with the engine stopped and under the influence of natural wind and waves, ⑤ denotes the zigzag maneuver as shown in Figure 7, and ⑥ denotes a sharp turn at a 360° circle. During ①–⑤, the liquid level detected by the five 500 mm long MgB_2_ level sensors at a heater input of 9 W was synchronously measured with the temperature and pressure inside the cryostat. Finally, during ⑥, the temperature and pressure were synchronously measured without the liquid level at a heater input of zero under natural heat-input conditions. All the rapid-depressurization tests were conducted by manually opening the release valve after achieving a pressure of around 0.2 MPaG. Tests ① and ⑥ were reported. Herein, we discuss Test ⑤ when the ship motion was maximum with liquid-level measurement.

## 4. Results and Discussion

**Figure 8:**
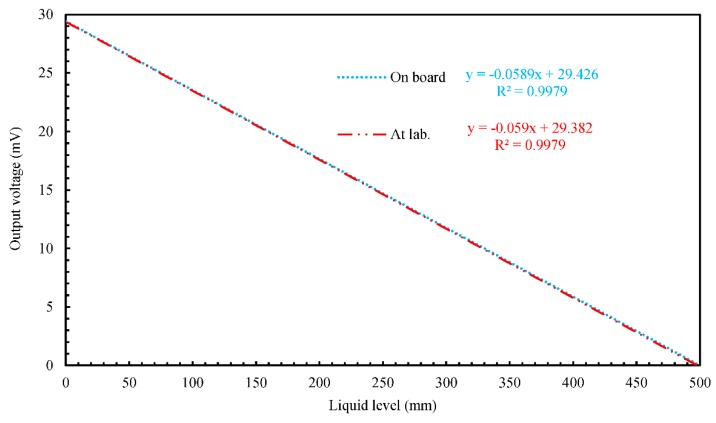
Comparison of onboard and laboratory results for the static level-detecting characteristics of the 500 mm long MgB_2_ level sensor A1 at a heater input of 9 W.

To investigate the influence of disturbances, such as onboard noise, we examined the static level-detecting characteristics of the five 500 mm long MgB_2_ level sensors onboard. We did this by determining the relationship between the sensor output voltage and the liquid level at atmospheric pressure and a heater input of 9 W while we decreased the liquid level (i) from 130 to 85 mm for Sensors A1 and C, and (ii) from 100 to 55 mm for Sensors A2, B1, and B2. The liquid-level ranges observable from the optical windows were 85–130 mm at Sensors A1 and C, and 55–100 mm at Sensors A2, B1, and B2 because the vertical positions of the windows differed in the X and Y directions. In this experiment, we used a four-wire technique to measure sensor output voltage. The onboard and laboratory [11] results are compared in Figure 8. This figure shows the linear approximate curved line except the experimental result for ease of viewing. The gradients of the straight lines approximating the onboard and laboratory data agree with each other, as do the relevant Y intercepts. No influence of disturbances can be seen in Figure 8. The results from the other sensors show similar tendencies.

**Figure 9:**
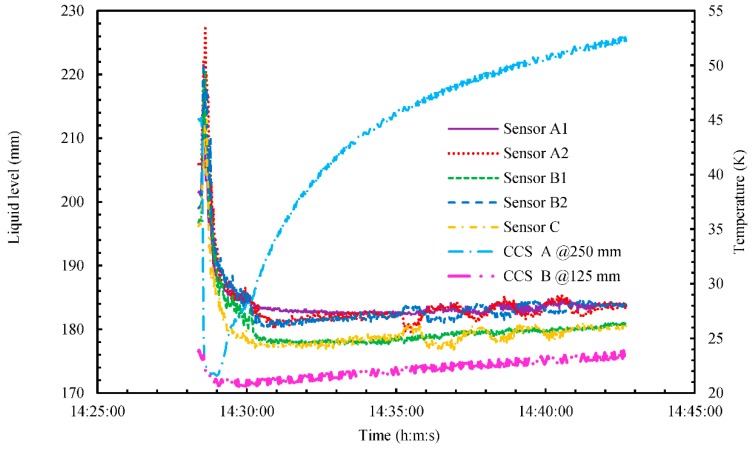
Time series of liquid level and temperature inside the cryostat during marine-transportation test ⑤ from 14:28 to 14:42.

**Figure 10:**
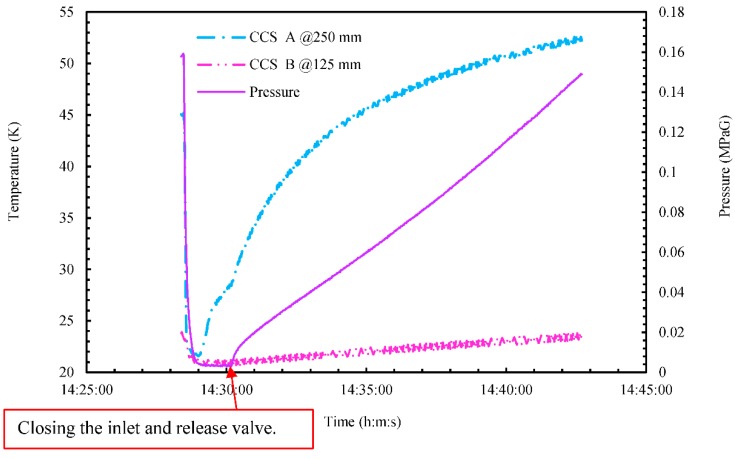
Time series of temperature and pressure inside the cryostat during marine-transportation test ⑤ from 14:28 to 14:42.

**Figure 11:**
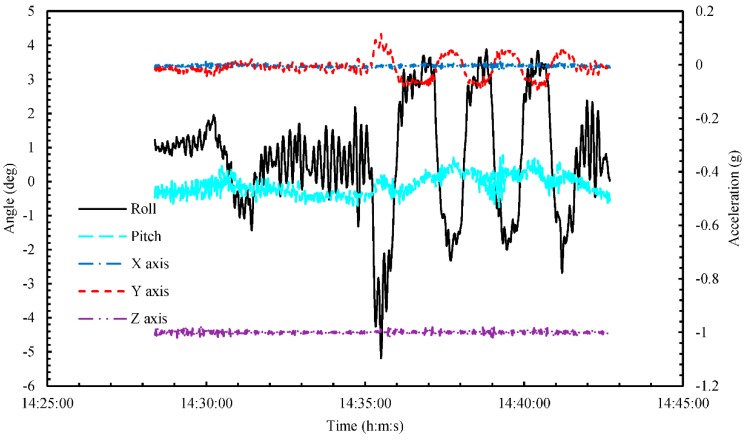
Time series of angle and acceleration during marine-transportation test ⑤ from 14:28 to 14:42.

Figure 9, Figure 10 and Figure 11 show the experimental results for liquid level, temperature, pressure, ship motion, and accelerations during marine-transportation test ⑤ from 14:28 to 14:42 at 1 s intervals. As shown in Figure 9, the liquid level increased rapidly from around 200 to 220 mm, as indicated by all five MgB_2_ level sensors. This was due to bumping of the LH_2_ surface, with the temperature of CCS A at the position of the gaseous phase rapidly decreasing, from around 45 to 21 K after 14:28. This is because mist occurred in the LH_2_ space. This phenomenon was observed by the naked eye.

At the same time, pressure rapidly decreased from around 0.16 MPaG, as shown in Figure 10. This is because the rapid-depressurization tests were conducted by opening the release valve after 14:28. The pressurization test was started at 14:30 by closing the release valve, as shown in Figure 10. During the zigzag maneuver after 14:35, all five MgB_2_ level sensors detected sloshing inside the small LH_2_ tank, as shown in Figure 9. At the same time, as shown in Figure 11, the ship moved with a maximum rolling angle of around 5°, a maximum pitching angle of around 0.8°, and a maximum Y-direction (i.e., the roll direction) acceleration of roughly 0.1 g, where g is acceleration due to gravity. In this experiment, the rolling and pitching angles were small because the experimental area was an inland sea in Osaka Bay. By comparison, the maximum rolling and pitching angles of the training ship on the open sea are around 25° and 20°, respectively. During the zigzag maneuver (14:35–14:41), pressure increased to roughly 0.67 MPaG/h, and CCS A temperature increased to roughly 1.0 K/min, as shown in Figure 10. By comparison, for about zero rolling and pitching angles (Tests ①–④), pressure increased to roughly 0.45 MPaG/h and CCS A temperature increased to roughly 2.2 K/min. We reason that the higher rate of pressure during the zigzag maneuver was due to sloshing inside the small LH_2_ tank, which increased the amount of BOG as heat was exchanged with the inner wall of the tank. On the contrary, the lower rate of the temperature of CCS A during the zigzag maneuver was due to cooling GH_2_ inside the small LH_2_ tank by the amount of BOG.

In the case of all five external heater input values being 0 W (without liquid-level measurement), pressure increased to roughly 0.15 MPaG/h and CCS A temperature increased to roughly 1.1 K/min with a maximum rolling angle of six degrees, and a maximum pitching angle of two degrees in the Test ⑥ [14].

In addition, the temperature of CCS B located within liquid phase increased from 22 to 24 K with the increased pressure after 14:30. This means that the saturation temperature of the LH_2_ increased with increasing the pressure of the small LH_2_ tank.

**Figure 12:**
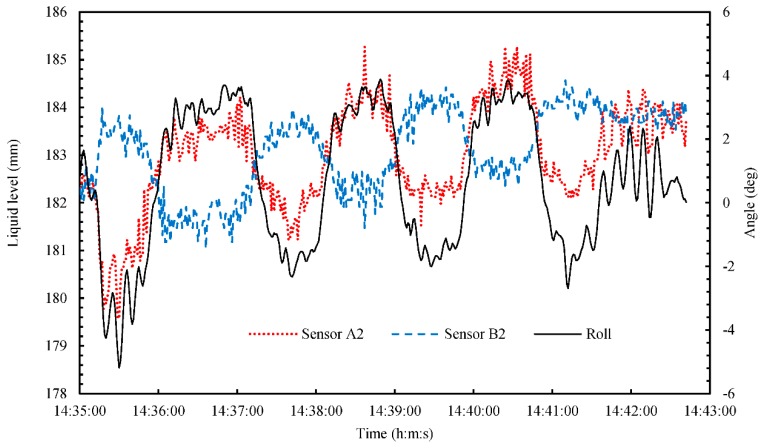
Time series of the liquid level and rolling angle during a zigzag maneuver from 14:35 to 14:42.

**Figure 13:**
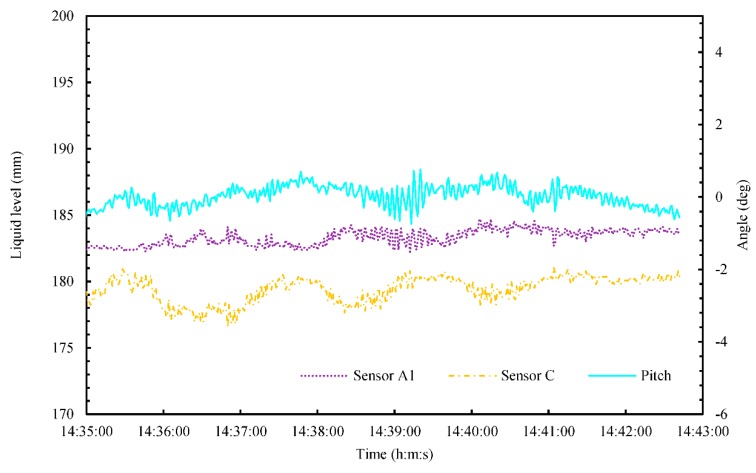
Time series of the liquid level and pitching angle during a zigzag maneuver from 14:35 to 14:42.

Figure 12 shows the time series of the liquid level and rolling angle during the zigzag maneuver (14:35–14:42). In this figure, the error bar of liquid level and rolling angle were omitted to understand the time variation. To clarify the relationship between liquid level and rolling angle, we used the rolling angle and the results from Sensors A2 and B2. As shown in Figure 12, the rolling signal was in phase with that of Sensor A2, and out of phase with that of Sensor B2. The average rolling period was 114 s, and the average liquid oscillation period was 118 s at the positions of Sensors A2 and B2. Figure 13 shows the time series of the liquid level and pitching angle during the zigzag maneuver (14:35–14:42). In this figure, the error bar of liquid level and pitching angle were omitted to understand the time variation. To clarify the relationship between liquid level and pitching angle, we used the pitching angle and results from Sensors A1 and C. As shown in Figure 13, the Sensors A1 and C can be detected the small liquid oscillation with small pitching angle.

## 5. Summary

The first experiments on LH_2_ marine transportation by the training ship Fukae-maru were carried out successfully with the measurement of the liquid level inside the small LH_2_ tank using five 500 mm long external-heating-type MgB_2_ level sensors. We synchronously measured the liquid level, temperature, pressure, ship motion, and accelerations during a zigzag maneuver. It was found that sloshing inside the small LH_2_ tank affected the rates of temperature and pressure increase during the zigzag maneuver, for which the maximum rolling angle was 5°. Moreover, the level sensors detected a long-period LH_2_ wave due to the ship’s motion. In this experiment, the rolling and pitching angles remained small because the experimental area was an inland sea in Osaka Bay. As future work, to clarify the influence of sloshing inside an LH_2_ tank in rough seas, we plan to perform experiments in the open sea.

## Figures and Tables

**Table 1:** Time chart of experimental processes conducted inside Osaka Bay.

Time	Process	Test Number
12:53	Sail out of port	①
13:02	Release valve close	
13:22	Release valve open (rapid depressurization)	
13:25	Release valve close	②
13.42	Release valve open (rapid depressurization)	
13:45	Release valve close	③
14:02	Release valve open (rapid depressurization)	
14:10	Release valve closed	④
14:10	Start drifting after stopping engine	
14:17	Finish	
14:28	Release valve open (rapid depressurization)	⑤
14:30	Release valve closed	
14:35	Zigzag maneuver test	
14:42	Finish	
14:45	A sharp turn at 360-degree circle	⑥
14:49	A ninety-degree sharp turn to the left	
14:51	Finish	
14:55	A sharp turn at 360-degree circle	
14:58	Finish	
15:58	A sharp turn at 360-degree circle	
16:17	Sail in port	
